# Supplementary material for: Quantitative optical nanophysiology of Ca2+ signaling at inner hair cell active zones
Source: Nat Commun. 2018 Jan 18;9:290. doi: 10.1038/s41467-017-02612-y (PMC5773603; doi:10.1038/s41467-017-02612-y)
Supplement: Supplementary file 1 — Supplementary Information [file 41467_2017_2612_MOESM1_ESM.pdf]

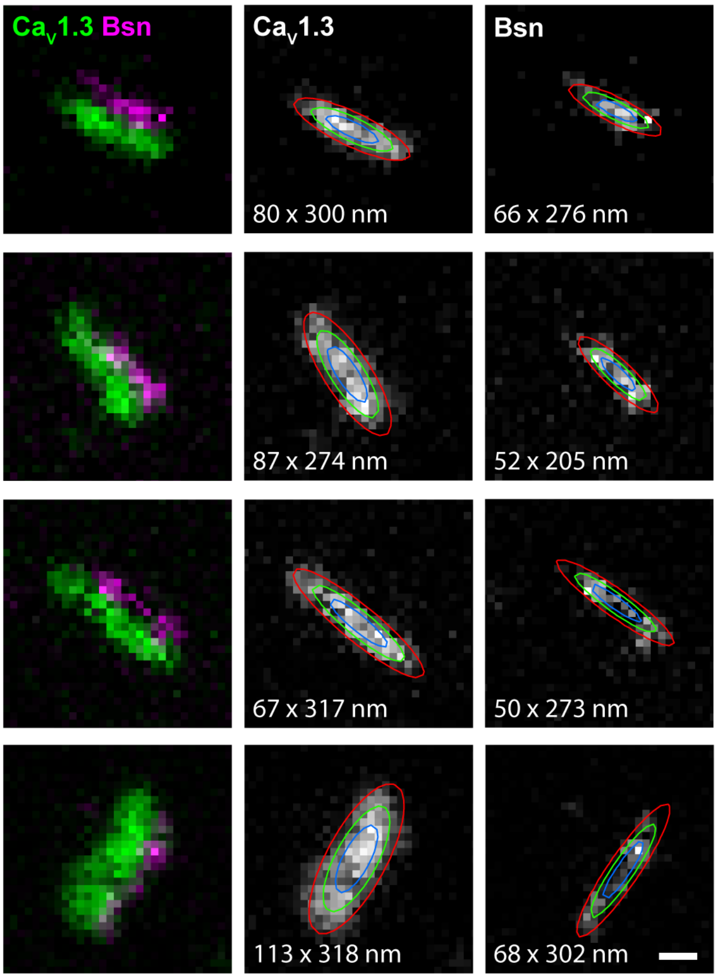

Supplementary Figure 1: Measuring the size of Ca^{2+}-channel- and bassoon-clusters using 2D Gaussian fits

The lengths and widths of $Ca_V1.3$ Ca^{2+}-channel- and bassoon-clusters were measured by fitting 2D STED images of the $Ca_V1.3$ (green) and bassoon (magenta) immunofluorescence at IHC AZs using 2D Gaussian functions. The resulting fit profiles are indicated by line contours, with green lines indicating the half-maximum. The resulting full-widths-at-half-maxima of the long and short axes are indicated below the fits. Scale bar: 100 nm.

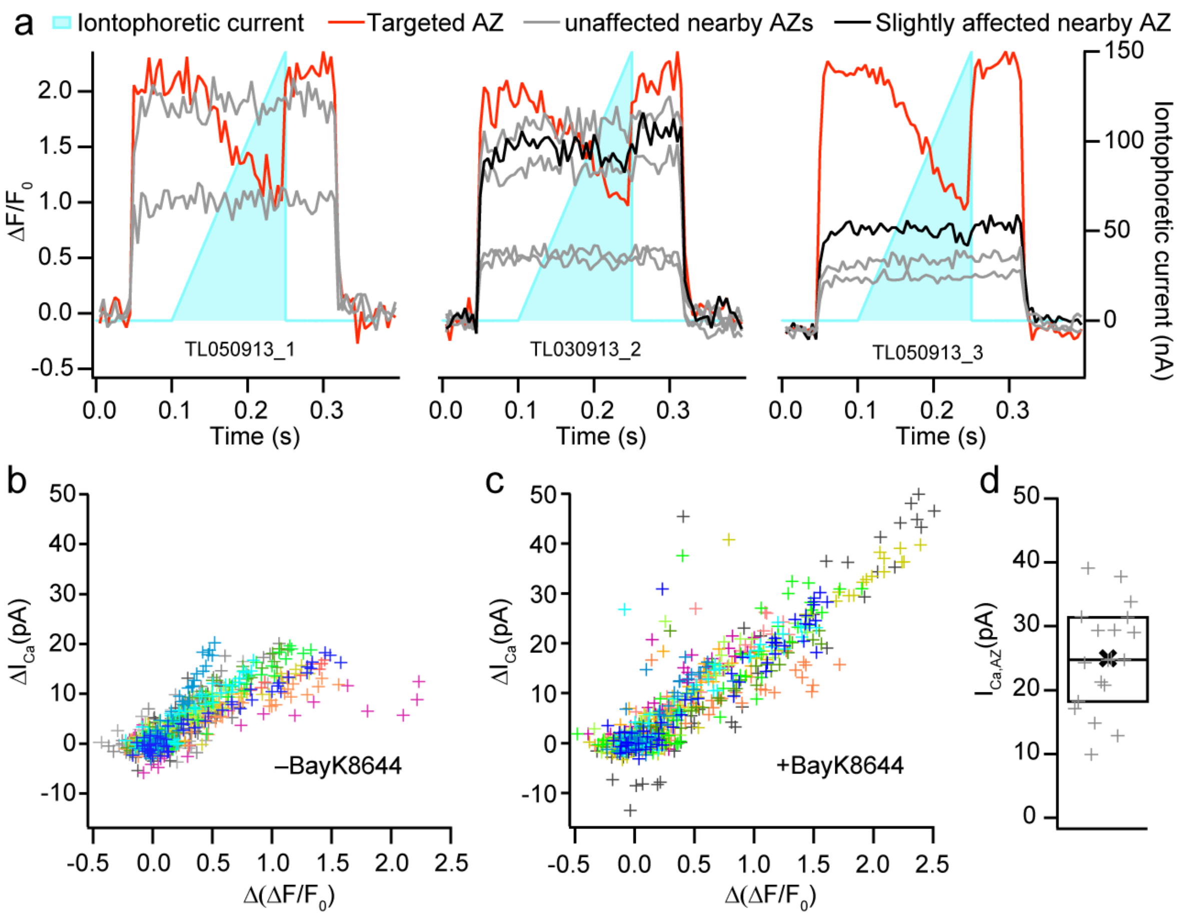

Supplementary Figure 2: Estimating synaptic Ca^{2+}-influx by combining focal depletion of extracellular [Ca^{2+}] with confocal Ca^{2+}-imaging of AZ Ca^{2+}-signals.

(a) Monitoring of the fluorescence change of targeted (red lines) and neighboring synapses (grey and black lines) during depolarization (from 0.05 to 0.32 s) and increasing iontophoretic application of EGTA (indicated by blue line) showed that while most neighboring synapses were virtually unaffected by the application (grey lines), some showed a minor decrease in $\Delta F/F_0$ (black lines). Fitting of a line to the part of the recording during which iontophoresis was active (0.1 to 0.25 s) allowed determination of the final change in $\Delta F/F_0$ of the neighboring synapses. This was then used to correct the change in I_{Ca}.

(b) Plot of the change of whole-cell Ca^{2+}-current (ΔI_{Ca}), evoked by iontophoretic application of EGTA, against the resulting $\Delta(\Delta F/F_0)$ at n=19 targeted AZs from N=19 IHCs. Different colors indicate data from different cells.

(c) Same as in (b), recorded in the presence of 5 µM of the agonistic dihydropyridine BayK8644 which prolongs the opening time of Ca^{2+}-channels, thus doubling the depolarization-evoked currents and $\Delta F/F_0$ (n=12 targeted AZs from N=12 IHCs).

(d) Box plot of the Ca^{2+}-currents carried by the individual AZs from (b), calculated from the depolarization-evoked change in AZ Ca^{2+}-indicator fluorescence ($\Delta F/F_0$) and the $(\Delta(\Delta F/F_0))/(\Delta I_{Ca})$-slope obtained from the change of synaptic Ca^{2+}-current during iontophoretic application of EGTA. The average value is indicated by a bold black cross, while individual data points are shown as small grey crosses.

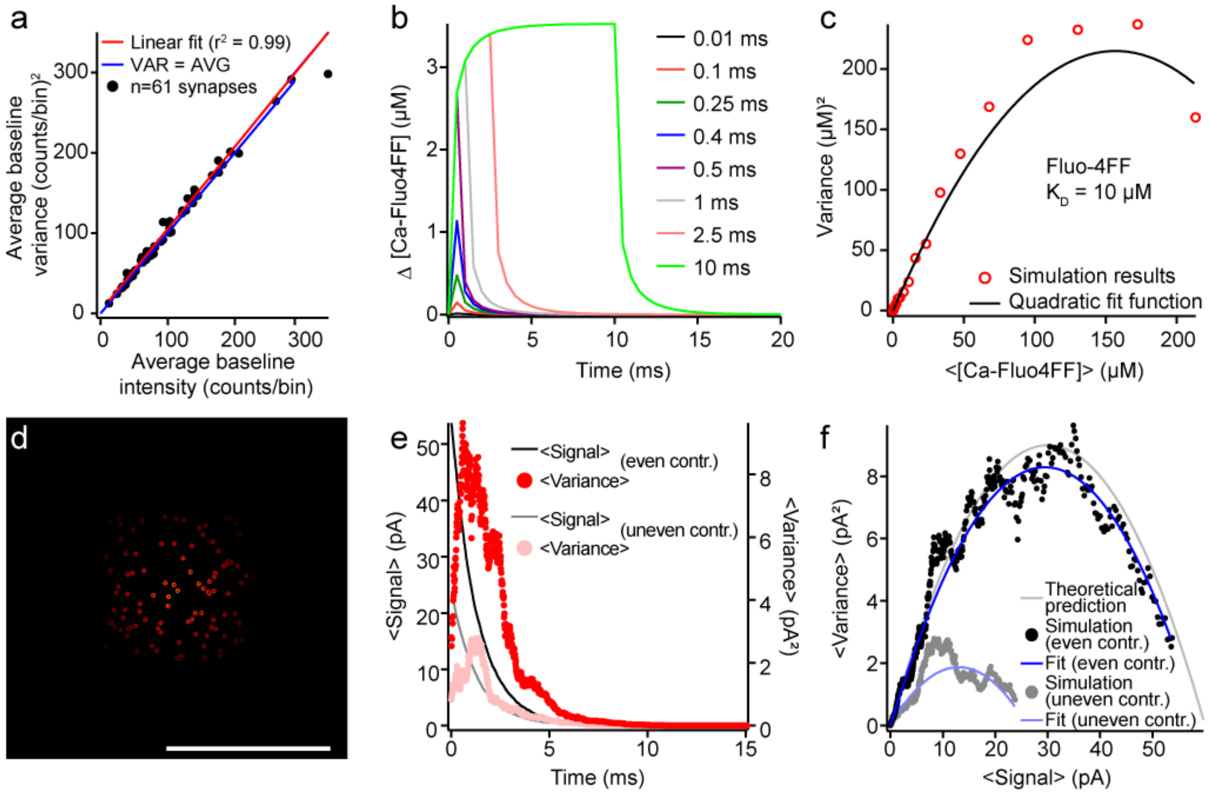

Supplementary Figure 3: Validating the estimation of the number of Ca^{2+}-channels at the AZ by optical fluctuation analysis

(a) Baseline variance (at -117 mV) of Fluo-4FF fluorescence is determined by photon shot noise ($\sigma^2 FCa \approx$ <FCa>).

(b) Simulated Ca^{2+}-binding responses of Fluo-4FF (Δ[Ca-Fluo4FF]) to varying open times of a single Ca^{2+}-channel (i_{Ca} = 0.6 pA) in center of PSF. The amplitude of the response to openings > 5 ms reaches a steady-state due to the balance of Ca^{2+}-influx and Ca^{2+}-removal by the non-fluorescent Ca^{2+}-chelator EGTA (10 mM). The growing amplitude with increasing open times for shorter openings is mainly due to the limited on-rate of the indicator ('sampling': 2 kHz; 1 mM Fluo-4FF, 10 mM EGTA).

(c) Example simulation with N_{Ca} = 100 (*equal contr. to signal; 50 sweeps*), using Δ[Ca-Fluo4FF]) responses (see (b)) to exponentially distributed closing times of the 100 Ca^{2+}-channels (τ = 1.3 ms). This simulation yielded N_{Ca} = 116. On average (100 simulation repetitions), N_{Ca} was estimated as 88 ± 22 (± SD). The bandwidth limitation imposed by the kinetics of Ca^{2+}-binding to the indicator has no major effect on the estimation of the number of channels.

(d) Schematic illustration of unequal contribution of Ca^{2+}-channels (red circles; distributed randomly over a quadratic area with d = 420 nm), due to convolution with the microscope's PSF. Scale bar: 500 nm.

(e) Resultant underestimation of both mean signal (no reaction with Ca^{2+}-indicator considered) and mean trial-to-trial variance (N_{Ca} = 100, i_{Ca} = 0.6 pA, 50 sweeps, 100 kHz).

(f) Corresponding variance-to-mean relationships and quadratic fits (equal contribution: N_{Ca} = 106, i_{Ca} = 0.56pA; unequal contribution: N_{Ca} = 92, i_{Ca} = 0.29 pA), as well as theoretical prediction (grey parabola). Note the underestimation of unitary response, but relative stable estimate of N_{Ca}.

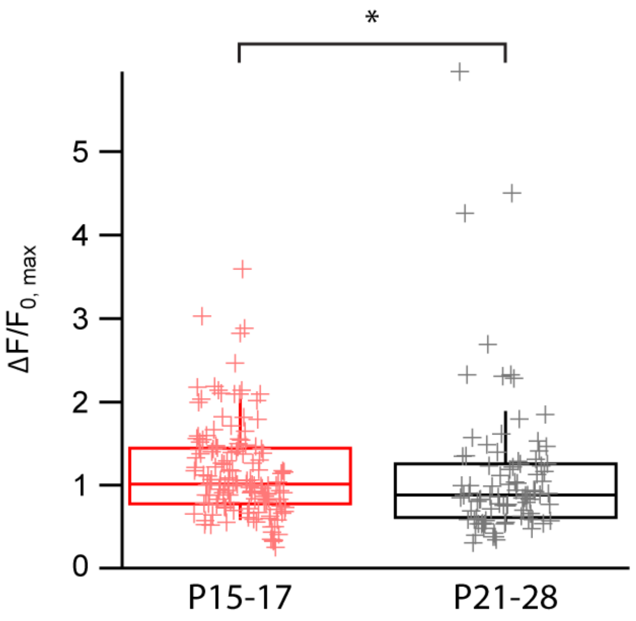

Supplementary Figure 4: Age-dependence of maximal presynaptic Ca^{2+}-increase

Synaptic Ca^{2+}-signals imaged by spinning-disk confocal microscopy as described for the selective suppression of Ca^{2+}-influx at individual AZs (Fig. 3). The box plot of the background-normalized maximal Fluo-8FF fluorescence increase ($\Delta F/F_{0,\ max}$) in response to ramp-depolarization of IHC active zones from wildtype mice shows a slight difference between age groups P15-17 (red, $\Delta F/F_{0,\ max}$=1.17 ± 0.05, median 1.02) and P21-28 (black, $\Delta F/F_{0,\ max}$=1.12 ± 0.09, median 0.88, p=0.03, Mann Whitney *U* test, statistical power: 0.07).

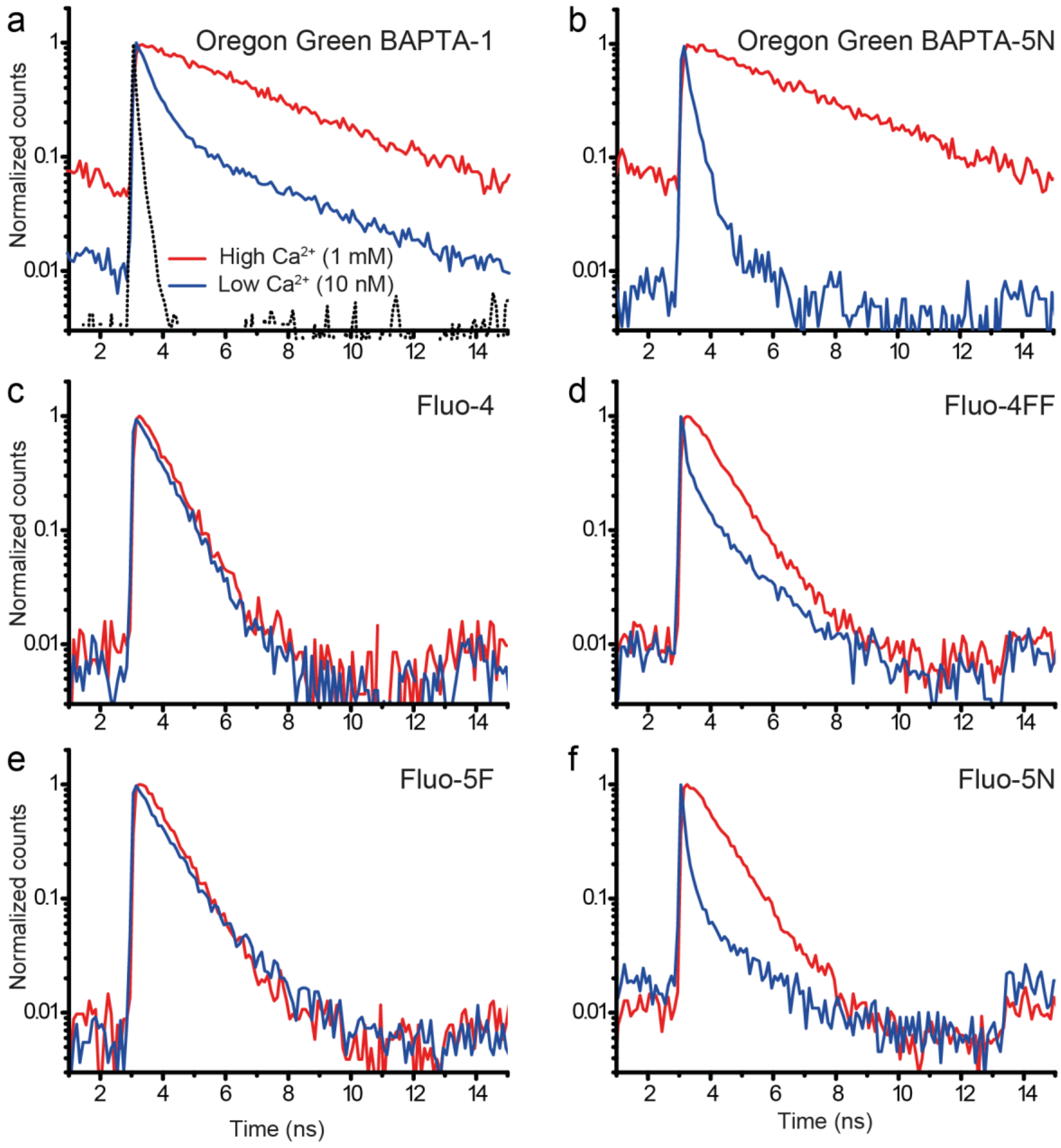

Supplementary Figure 5: Fluorescence lifetime measurement of various Ca^{2+}-indicators.

(a-f) The fluorescence lifetime of a selection of frequently used Ca^{2+}-indicators was recorded in confocal spot-detection with time-correlated single-photon counting at high and low [Ca^{2+}] in buffered saline (Calbuf-1, World Precision Instruments, Sarasota, FL, USA). The normalized log-normal plots show an increased lifetime upon Ca^{2+}-binding for Oregon Green BAPTA-1 (OGB-1; a), OGB-5N (b), Fluo-4FF (d) and Fluo-5N (f), while no lifetime change was found for Fluo-4 (c) and Fluo-5F (e) between high and low [Ca^{2+}]. All indicators show a linear decline in the log-normal plot form which indicates a mono-exponential decay. Dotted line in (a): Impulse response function. In conclusion, some but not all indicators that show a large Ca^{2+}-dependent increase in fluorescence display a Ca^{2+}-dependent change in lifetime. The Ca^{2+}-dependent fluorescence increase of Ca^{2+}-indicators can be due to a change in quantum yield (Ca^{2+}-dependent lifetime), a change in the extinction coefficient, or in both parameters[2].

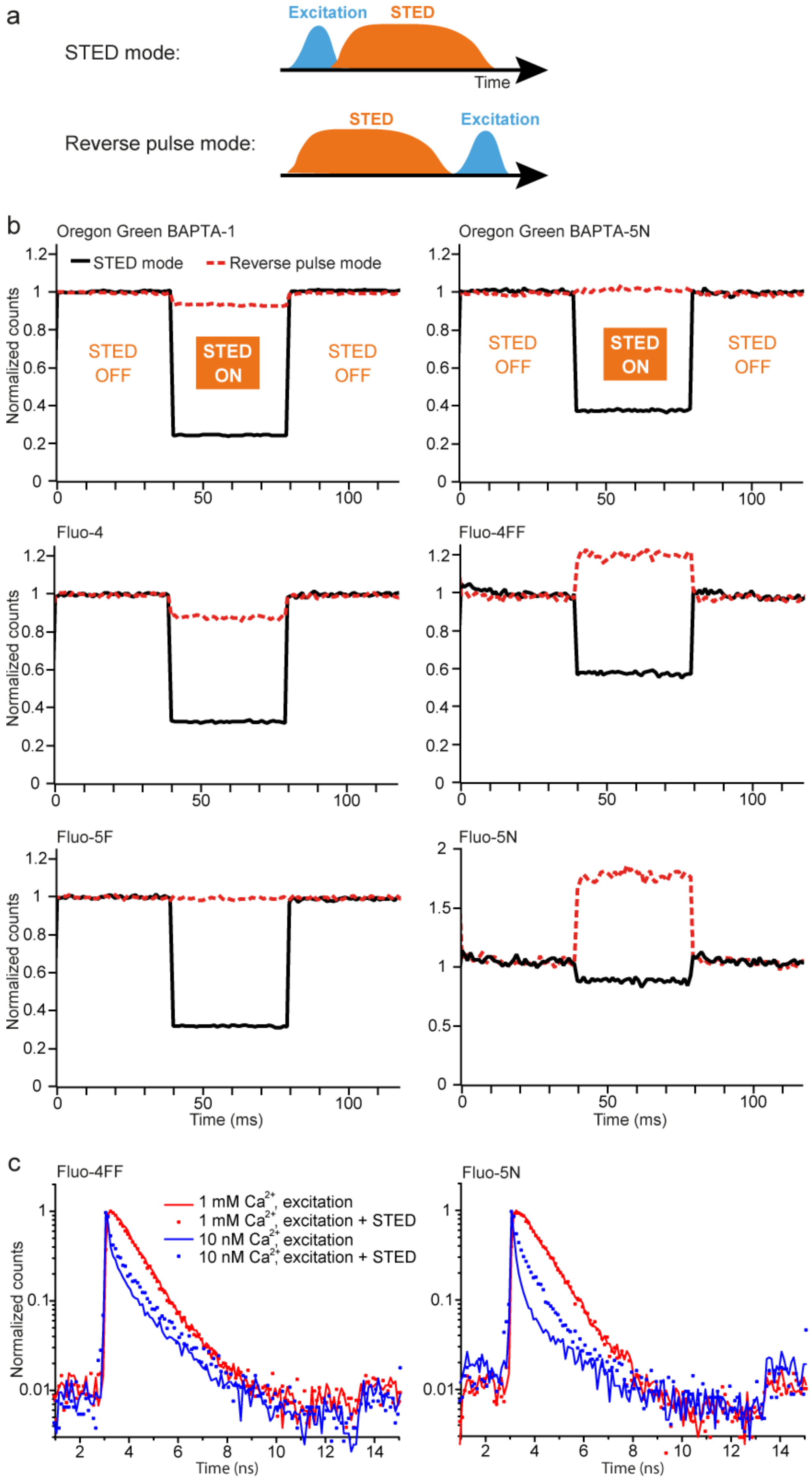

a
STED mode:
Excitation
STED
Time
Reverse pulse mode:
STED
Excitation
b
Oregon Green BAPTA-1
STED mode
Reverse pulse mode
STED OFF
STED ON
STED OFF
Normalized counts
Oregon Green BAPTA-5N
Fluo-4
Fluo-4FF
Fluo-5F
Fluo-5N
Time (ms)
c
Fluo-4FF
1 mM Ca2+, excitation
1 mM Ca2+, excitation + STED
10 nM Ca2+, excitation
10 nM Ca2+, excitation + STED
Fluo-5N
Time (ns)

Supplementary Figure 6: Photostability of fluorescent Ca^{2+}-indicators in STED microscopy.

(a) Indicators were dissolved in low Ca^{2+}-buffer solution (10 nM [Ca^{2+}]) and fluorescence was recorded at 488 nm excitation. Fluorescence was depleted with a Gaussian shaped STED beam at 595 nm. The STED beam was switched on and off with an electro-optic modulator. The only difference in STED mode compared to the Ca^{2+}-spot measurements was that the phase plate was removed to switch off the fluorescence as much as possible. In reverse mode the temporal alignment was changed; the excitation pulse of ~110ps full-width half-maximum was delayed and followed the ~300ps long STED pulse. Here, no change in fluorescence intensity would be expected due to the STED pulse if the only effect of the STED laser on the dye would be stimulated emission depletion. Effects of the STED laser in reverse mode, however, would point to potential photo-damage.

(b) STED switched off the fluorescence of the Ca^{2+}-indicators OGB-1, OGB-5N, Fluo-4, and Fluo-5F but had no or very little effect on the fluorescence in reverse pulse mode when the fluorophores were in the ground state. Fluo-4FF and Fluo-5N, however, showed an increase in fluorescence in reverse pulse mode in low [Ca^{2+}] which might result from photo-damage of the BAPTA chelator from the dye by the STED light.

(c) Lifetime measurement of Fluo-4FF and Fluo-5N with and without STED in low and high [Ca^{2+}]. Due to the reverse pulse used here, no change in lifetime should be visible when using STED light. Both indicators showed a change towards longer fluorescent lifetime with STED light in low [Ca^{2+}] (dotted and straight blue line) while there was no change in high [Ca^{2+}] (red). The increase in lifetime of the Ca^{2+}-free dye might reflect photo-damage of the Ca^{2+}-chelator moiety of the dye which normally quenches the fluorescence. OGB-1, OGB-5N, Fluo-4 and Fluo-5F, showed no indication for photo-damage with lifetime imaging or with STED in reverse pulse mode (b) and therefore are suitable for STED Ca^{2+}-imaging. Our study thereafter focused on using OGB-5N because its low affinity met our requirements for imaging AZ Ca^{2+}-signals.

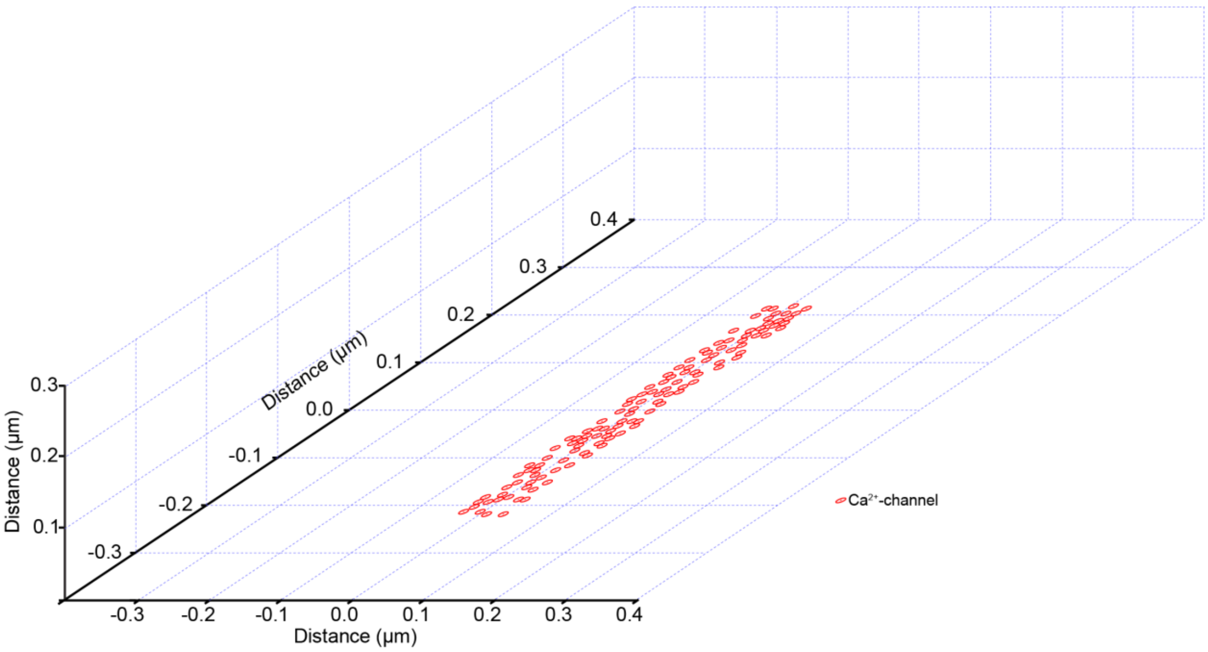

Supplementary Figure 7: Layout of the theoretical model of AZ topography.

120 Calcium channels were randomly distributed across a 67 nm x 430 nm rectangular area, with a nearest-neighbor-distance of at least 10 nm.

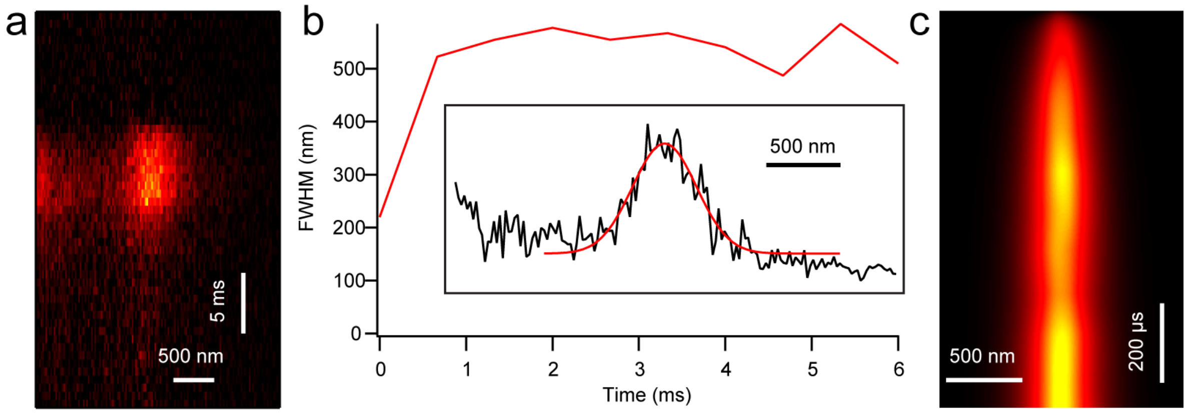

Supplementary Figure 8: Lateral spread of fluorescence from IHC AZs

(a) Average of 38 line scans of fluorescence increase at an IHC AZ evoked by short (5 ms) depolarizations to -14 mV.

(b) Full width of half maximum of Gaussian fits to individual lines from the recording shown in (a). The inset shows a representative fit (red) on top of the line profile (black). Note that the size of the Ca^{2+}-domain reaches a steady state within less than 1 ms.

(c) Mathematical simulation of the increase of Ca^{2+}-bound OGB-5N at an IHC AZ during a 1 ms depolarization, from the model shown in Fig. 5.

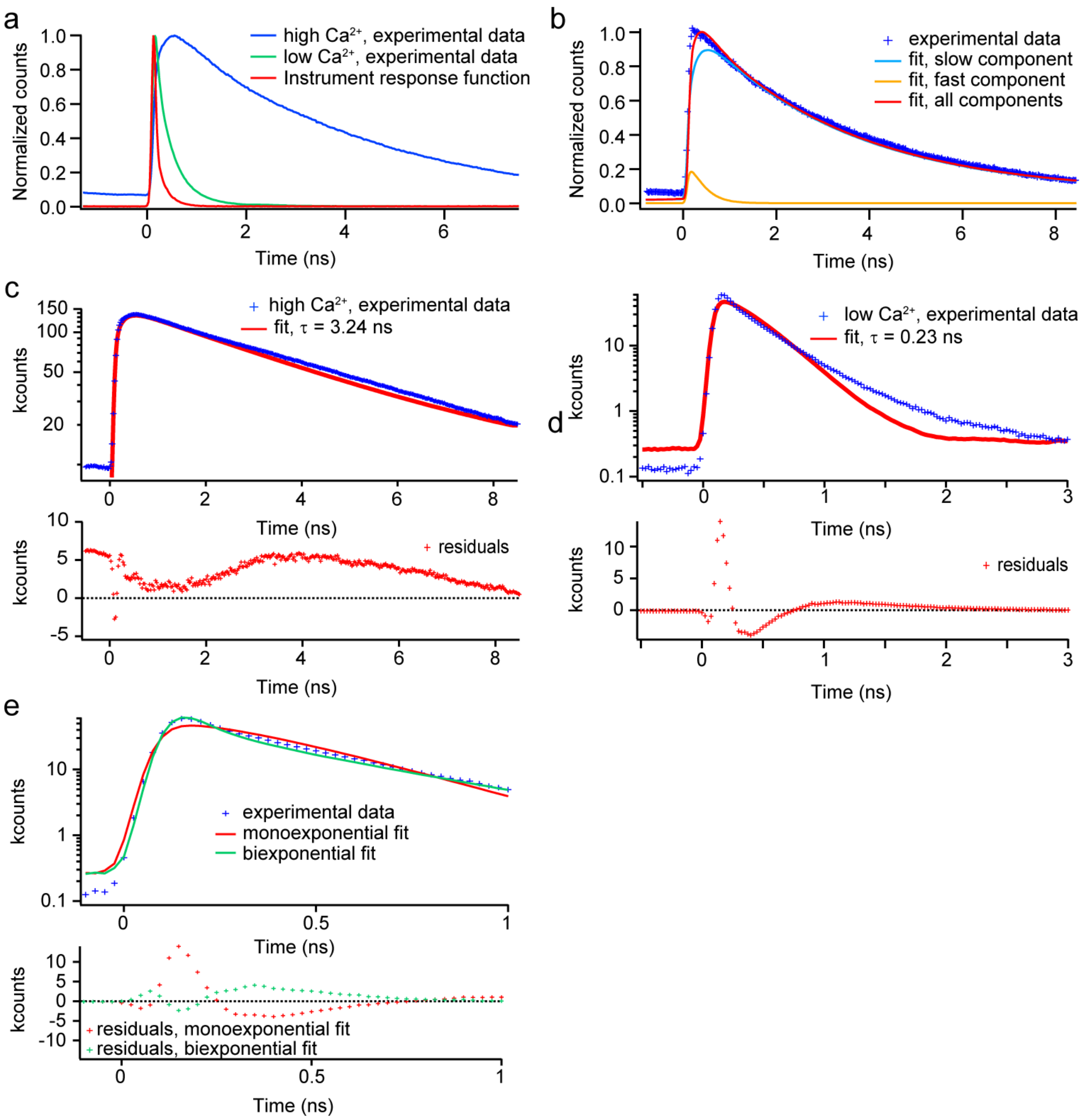

Supplementary Figure 9: Analysis of OGB-5N lifetime recordings: Fitting procedure and instrument response function

(a) The two possible states of the Ca^{2+}-indicator dye OGB-5N, Ca^{2+}-bound (blue trace) and Ca^{2+}-free (green trace), exhibit starkly different fluorescence lifetimes and extinction coefficients and therefore can be discerned readily by fluorescence decay analysis. The total fluorescence signal from OGB-5N at an arbitrary Ca^{2+}-concentration will be a superposition of fluorescence from both states, and can be fitted by a bi-exponential function convolved with the instrument response function of the microscope (IRF, red trace), which was recorded by putting a mirror in the object plane.

(b) An exemplary confocal fluorescence decay recording (blue crosses) was fitted with an IRF-convolved biexponential function (red trace), allowing the faithful dissection of the OGB-5N signal into a slow (blue line) and a fast component (orange line).

(c, d) The fluorescence lifetimes of the Ca^{2+}-bound and Ca^{2+}-free state were determined by measuring the fluorescence decay of OGB-5N in either Ca^{2+}-saturated (c) or Ca^{2+}-free solution (d). The resulting data (blue crosses) were fitted with a single exponential function convolved with the system IRF (red), resulting in lifetimes of τ_B = 3.24 ns ± 0.03 ns for Ca^{2+}-bound OGB-5N (c) and τ_U = 0.23 ns ± 0.03 ns for free OGB-5N (d). The lifetime values of each state were held constant when fitting experimental data with unknown [Ca^{2+}], leaving only the amplitudes as free variables.

(e) The fluorescence decay of OGB-5N in Ca^{2+}-free solution could not perfectly be described with a monoexponential decay, with the fit deviating perceptibly beyond 1 ns. By adding a second exponential decay component with a longer lifetime (between 0.3—1.0 ns) to the fit, the later deviations could be removed fairly well. Noticeably, however, this did not significantly improve the fit during the initial first nanosecond. Furthermore, the amplitude of the additional (longer) lifetime component was smaller by about 1—2 orders of magnitude, and the lifetime of the second component varied unpredictably between different measurement trials. Conclusively, we did not add this third decay component to the fitting procedure for evaluating experimental data, as it added high unpredictability to the fits without noticeably improving the quality or robustness of the results.

Additional notes:

When fitting experimental data, the long and short lifetime values were kept constant, as predetermined by calibration measurements. The resulting amplitudes of the bi-exponential fit (α_B and α_U, respectively) were then normalized using the total (fluorescence) photons F, according to the identity:

$$F = F_{\mathrm{B}} + F_{\mathrm{U}} = \alpha_{\mathrm{B}}\tau_{\mathrm{B}} + \alpha_{\mathrm{U}}\tau_{\mathrm{U}} \text{ (Equation 18)}$$

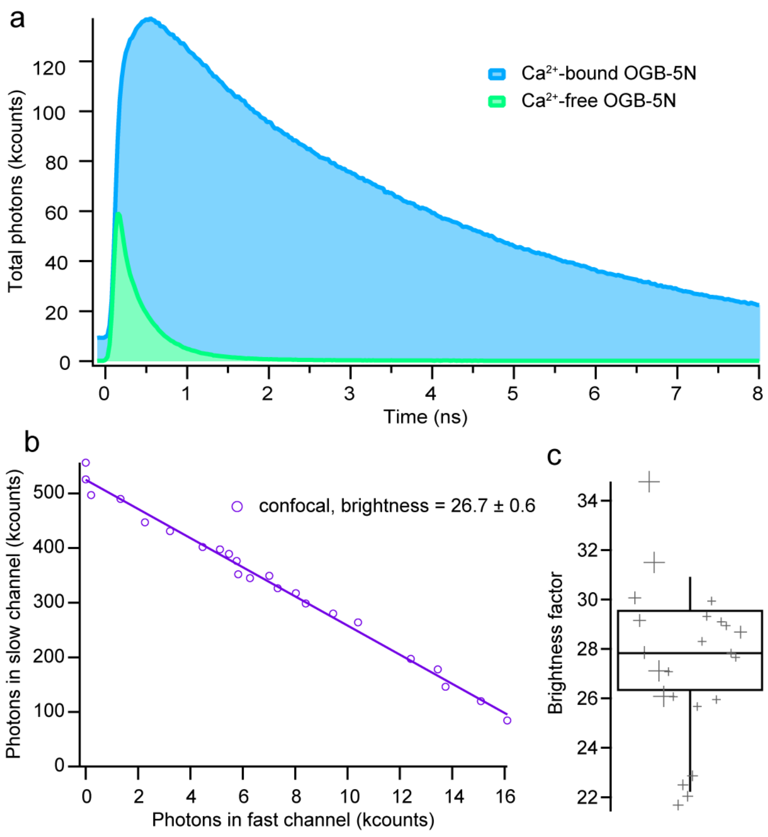

Supplementary Figure 10: Estimation of the brightness increase factor *b*

(a) When OGB-5N is not bound to Ca^{2+}, the brightness of the fluorophores is significantly diminished due to quenching. This means that for identical dye concentration under identical excitation and detection parameters, Ca^{2+}-bound OGB-5N (blue) emits many more photons than free OGB-5N (green). Displayed are fluorescence decay curves of OGB-5N recorded in saturating or desaturating Ca^{2+}-environments, the area under the curve denoting the detected photons.

(b) We corrected for this difference in brightness by determining the 'brightness increase factor' b, which denotes the increase in photons emitted by OGB-5N upon binding of Ca^{2+}. There are numerous ways to determine the brightness increase factor b, such as plotting the photons detected in the slow channels versus the fast channel and determining the slope of the linear fit to the data (here, 26.7 ± 0.6).

(c) We considered all measurements of the brightness increase factor *b* by combining them into a weighted average of the brightness increase of OGB-5N upon binding of Ca^{2+}, resulting in a brightness factor of *b* = 28.02, which was used for further calculations. Grey symbols are individual data points, size scaled according to the weighting factor which was assigned based on the number of individual measurements and the number of different samples used for each data point. The horizontal black line indicates the median of 27.83. A second method of determining the brightness increase was to measure the amplitudes of the bi-exponential fits determined by the fitting routine for the fluorescence decay curves, which resulted in an amplitude ratio (bound/unbound) of 1.99±0.06. Multiplication of this amplitude ratio with the ratio of fluorescence lifetimes $\frac{\tau_{slow}}{\tau_{fast}}$ results in a brightness factor of 28.0.

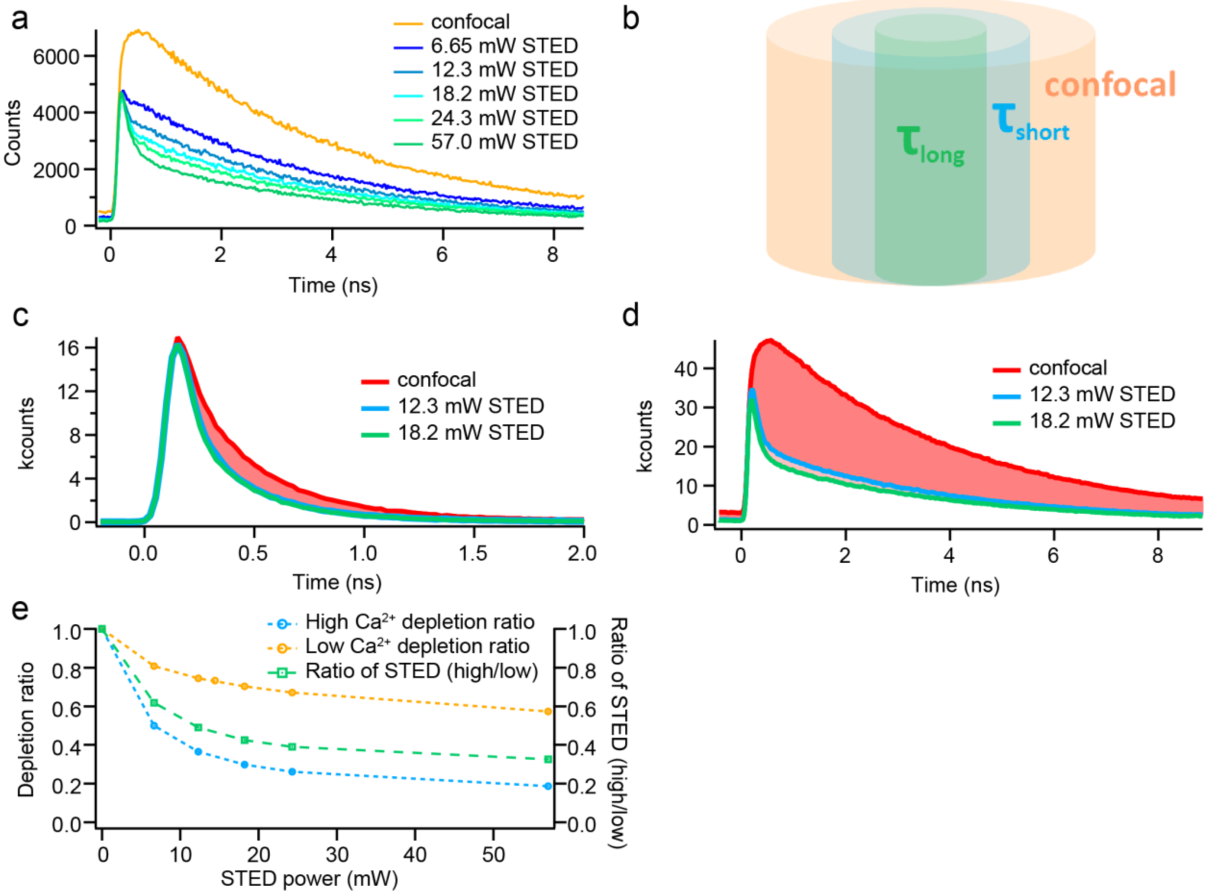

Supplementary Figure 11: Correcting for differences in the STED efficiency for Ca^{2+}-free and -bound OGB-5N

(a) The effect of increasing STED powers on the fluorescence signal recorded from Ca^{2+}-saturated OGB-5N dye solution. The STED beam induces a rapid de-excitation of excited fluorophores, leading to an additional fast fluorescence decay in the first 200–400 ps. After the STED pulse, the fluorescence decay returns to its natural lifetime of 3.24 ns.

(b) Identical STED powers achieve different effective focal volumes of the Ca^{2+}-bound and Ca^{2+}-free OGB-5N dye, due to different STED efficiencies. Sketch of the different effective focal volumes achieved by STED imaging of Ca^{2+}-bound (τ_{long}, green) and Ca^{2+}-free (τ_{short}, blue) OGB-5N compared to the focal volume from confocal imaging (orange). Not drawn to scale.

(c, d) The Ca^{2+}-bound dye (d) can be de-excited with STED much more efficiently than Ca^{2+}-free dye (c), primarily due to its longer lifetime. The difference in STED efficiency of the two states of the dye can be seen by comparing the reduction in fluorescence from confocal recordings (red) to measurements with moderate STED power (blue, green) for either in Ca^{2+}-free (c) or Ca^{2+}- saturated dye solution (d). The light red shaded areas indicate the difference in photon yield between confocal and STED recordings.

(e) Increasing STED power causes stronger reduction in fluorescence for Ca^{2+}-bound dye (blue, approximately 80% at 60 mW) compared to Ca^{2+}-free dye (orange, approximately 40% at 60 mW). The ratio of these STED efficiencies $s = \frac{d_{bound}}{d_{free}}$ (green, Equation 19) likewise denotes the ratio of effective focal volumes of the Ca^{2+}- bound and free dye. We used this ratio s to perform the STED efficiency correction while calculating [Ca^{2+}] from raw photon data, as described in the methods.

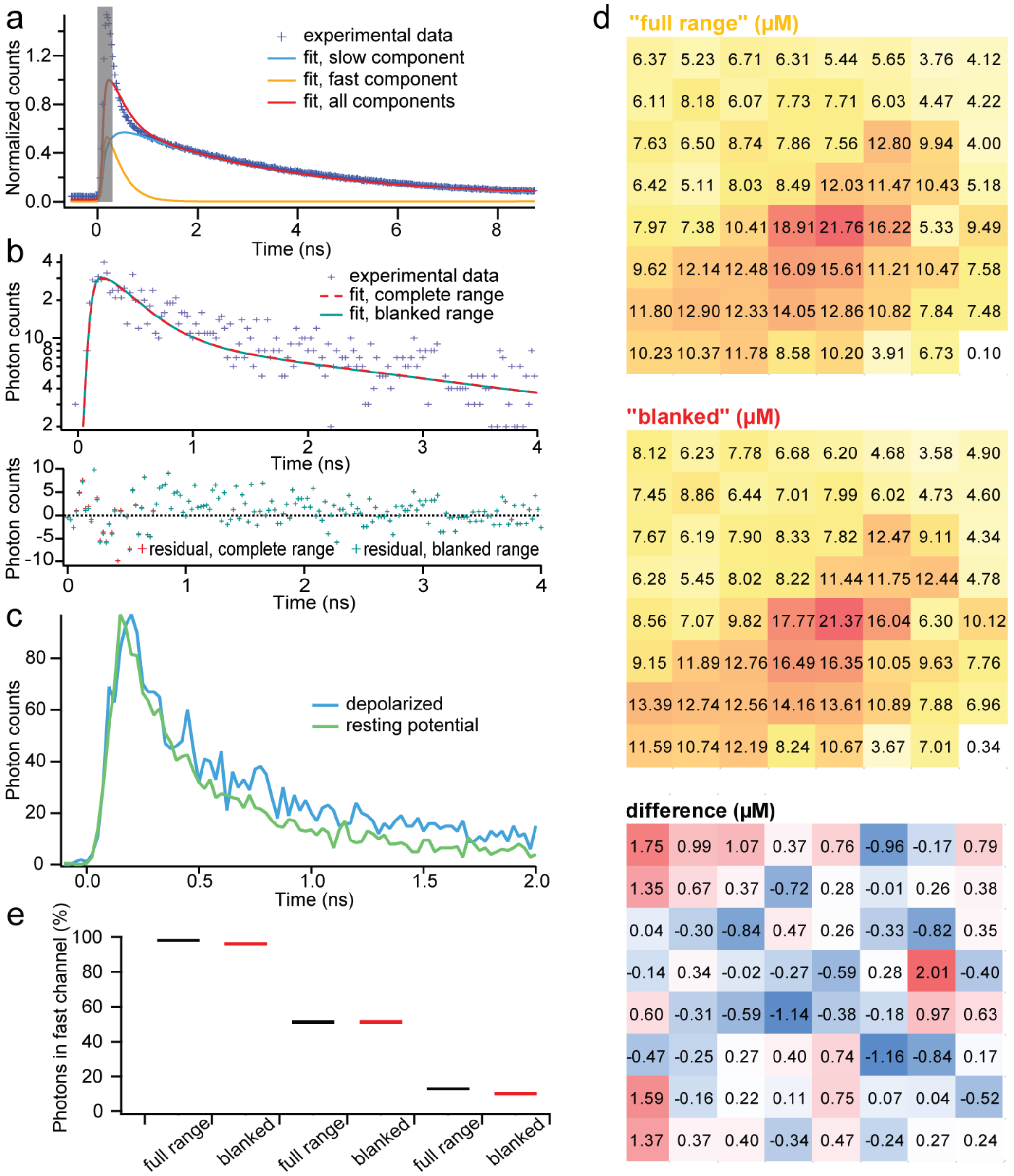

Supplementary Figure 12: Modification to the fitting procedure ("blanking") when acquiring fluorescence decay curves using STED nanoscopy

(a) The active STED beam causes a rapid fluorescence decay due to the de-excitation of fluorophores. In order to minimize the impact of this effect on the measured lifetime ratio, the time during which the STED beam was active (i.e. the first 300 ps after the onset of the excitation pulse) was excluded from the fit (grey bar). For calculating the fractions of slow and fast fluorescence, the amplitudes resulting from the fitting procedure were back-extrapolated to the onset of fluorescence, before being normalized using the total number of detected photons F.

(b) In order to test whether the "blanking" of the lifetime fits influenced the calculated [Ca^{2+}] values, we used experimental data (blue crosses) from confocal recordings (where blanking is not required) and fitted them using either the entire data range (red line) or the truncated range (green line, excluding the blanked range). This was done for data sets with different brightness levels and, different [Ca^{2+}] ranges, both depolarized and at resting potential. In general, "blanking" the initial

data range hardly altered the resulting fit at all, irrespective of brightness level or [Ca^{2+}]. The fit residuals (lower panel) for both methods were almost identical.

(c) One reason the fit algorithm is so robust, even when the initial range of the data is blanked, might be explained by the different brightness of the bound and unbound dye. Displayed are fluorescence decay curves recorded at a synaptic active zone, during depolarization (blue) and at resting potential (green). Note that the initial peak is nearly identical in height, but that the subsequent tail is notably higher for the depolarized curve. The blanking will not influence the algorithm fitting the long-lasting fluorescence decay, where the change in fluorescence due to the Ca^{2+}-binding of the dye is most noticeable.

(d) An exemplary confocal xy-raster recording at a synaptic active zone. Displayed are the resulting [Ca^{2+}] levels (in µM), as estimated by fitting either the entire data range (top panel) or the truncated data range (center panel, excluding the blanked range). The difference in calculated [Ca^{2+}] between both recordings (lower panel) showed no systematic bias, and on average the (absolute) [Ca^{2+}]-differences between both methods were less than 0.4 µM – 1 µM, depending on the [Ca^{2+}]-level recorded at the AZ. This corresponded to an average difference of approx. 2%–3% of the maximum [Ca^{2+}].

(e) The robustness of the blanked versus the full-range fits can also be illustrated by considering how many photons were sorted differently by the blanked fitting procedure. Displayed are the percentage of photons F_U/F assigned to the fast (unbound) channel, when fitting either the full range (black) or excluding the first 300 ps (red), for [Ca^{2+}] values of 10 nM, 11 µM, and 40 µM and for N = 50 identical measurements each. On average the amount of photons that were assigned differently when using the reduced fitting range was between 0.4—2.8%.

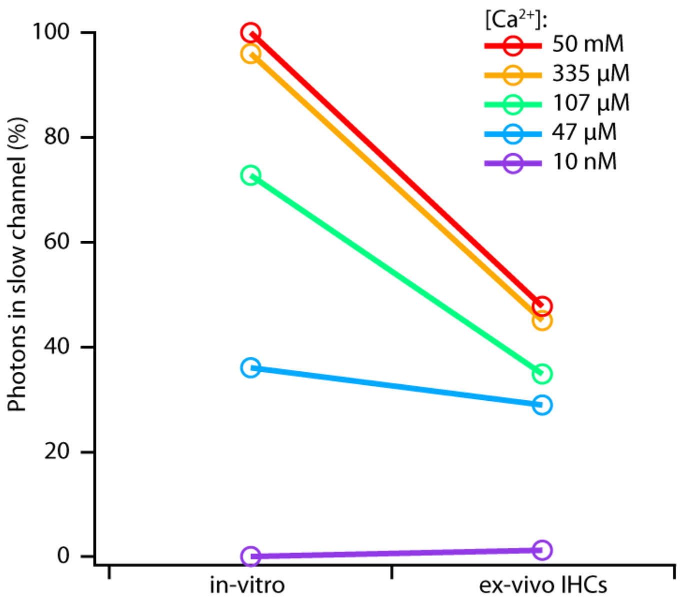

Supplementary Figure 13: *Ex vivo* calibrations in living IHCs

When measuring calibration solutions with different levels of [Ca^{2+}] *in vitro*, we were able to either completely populate (>99%) or depopulate (<1%) the Ca^{2+}-bound state of OGB-5N, allowing for precise calibration measurements of various dye properties, as well as determining [Ca^{2+}] with high confidence. When we tried to replicate these calibration measurements in *ex vivo* conditions, by filling IHCs with intracellular solution buffered to specific [Ca^{2+}] values, we noticed that the dye behaved differently. Despite filling the cells with highly saturating intracellular solutions (extremely high: [Ca^{2+}]= 50 mM (red) or very low: 10 nM (purple), respectively), we could neither depopulate the bound state below 1—1.5% (purple), nor populate the bound state to higher than ~55% (red). When measuring these exact same solutions either directly in the glass pipette or (blown out into) the extracellular solution, we were again able to measure a much higher dynamic range. It appears that inside the cells a certain fraction of the dye is unresponsive to the [Ca^{2+}] environment, showing either a permanently bound or unbound behavior. For this reason we considered this fraction of the dye to be [Ca^{2+}]-insensitive, and have included this fraction into our models to be able to re-calibrate the resulting [Ca^{2+}] values from the fluorescence decay measurements.

In conclusion, we estimate the maximum obtainable fraction of the signal in the slow channel to be ~60% and the minimum ~1%.

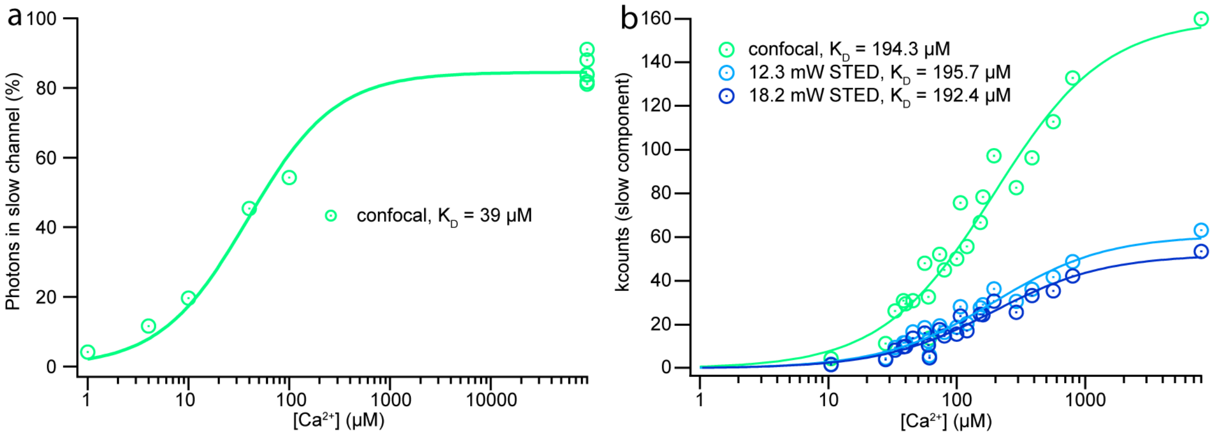

Supplementary Figure 14: In-vitro determination of the dissociation constant of OGB-5N

The dissociation constant K_D of the calcium indicator OGB-5N varied depending on the precise environment in which it was measured. We determined the effective dissociation constant K_{eff} of OGB-5N by recording the fluorescence decay of OGB-5N in commercially available Ca^{2+}-buffer (a) and in intracellular solution (b, used for patch-clamping) with various different Ca^{2+}-concentrations (ranging from 10 nm to 50 mM). The photon counts that were assigned to originate from the Ca^{2+}-bound dye (F_B) by the fitting routine were plotted against the calculated free $[Ca^{2+}]$ and fitted with either

$$R_B = \frac{([Ca^{2+}])}{([Ca^{2+}])+K_{eff}} \text{ (Equation 20)}$$

with R_B the fraction of Ca^{2+}-bound dye or

$$F_B = F_{B,max} \cdot \frac{([Ca^{2+}])}{([Ca^{2+}])+K_{eff}} \text{ (Equation 21)}$$

with $F_{B,max}$ being the maximum slow counts measured at saturating Ca^{2+}-concentration.

(a) In commercially available Ca^{2+}-buffer solutions (Calbuf-1 & Calbuf-2, WPI, Berlin, Germany) OGB-5N displayed a dissociation constant K_{eff} = 39 ± 4 µM, similar to values described previously.

(b) When we determined the effective dissociation constant K_{eff} of OGB-5N in an environment as close as possible to the *ex vivo* experiments, i.e. in intracellular solution (for patch-clamping) with various different Ca^{2+}-concentrations, we found the K_{eff} ~ 195 µM to be much higher both in confocal recordings (green data points and fit) and STED recordings using different STED intensities (blue and purple data points and fit).

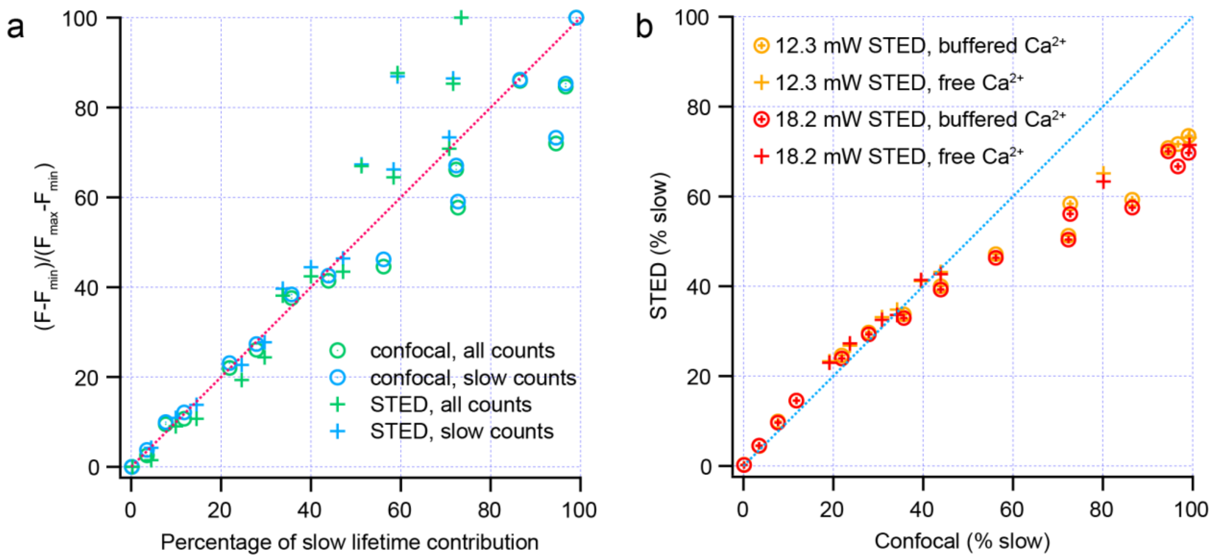

Supplementary Figure 15: Comparison of lifetime-based [Ca^{2+}] estimation to a fluorescence-increase based method - verification of STED lifetime measurements

(a) Comparison of the percentage of Ca^{2+}-bound dye, as determined by exponential fitting of fluorescence lifetime recordings for different [Ca^{2+}] to the established method of [Ca^{2+}] estimation by measuring the fluorescence increase upon Ca^{2+}-binding as

$$\frac{F-F_{\min}}{F_{\max}-F_{\min}} \text{ (Equation 22)}$$

resulted in a linear relation with a slope of 0.9 (confocal) or 1.3 (STED), irrespective of whether the calculation of $\frac{F-F_{\min}}{F_{\max}-F_{\min}}$ was performed with the total measured photon counts (green symbols) or only with those that had been assigned to the slow component of fluorescence decay (blue symbols). For lower Ca^{2+}-concentrations (less than 40% bound OGB-5N), the match was still better, with slopes of 1.0 (confocal) and 1.1 (STED).

(b) Comparing estimates of the percentage of Ca^{2+}-bound OGB-5N from STED and confocal spot detection for various [Ca^{2+}]. Estimates from measurements with dye in diluted pure $CaCl_2$ solution (crosses) and dye in intracellular solution with addition of Ca^{2+} and citrate (circles) showed a good agreement between confocal recordings and STED-recordings using different STED laser intensities (12.3 mW, yellow, and 18.2 mW, red), as long as Ca^{2+}-bound OGB-5N was lower than 40%. For higher percentages, STED imaging tended to overestimate the contribution of the fast component of fluorescence lifetime. All recordings in live IHCs resulted in Ca^{2+}-bound dye ratios of well under 40%, implying that both STED and confocal recordings allowed matching and faithful calculations of free [Ca^{2+}] in the our current experiments.

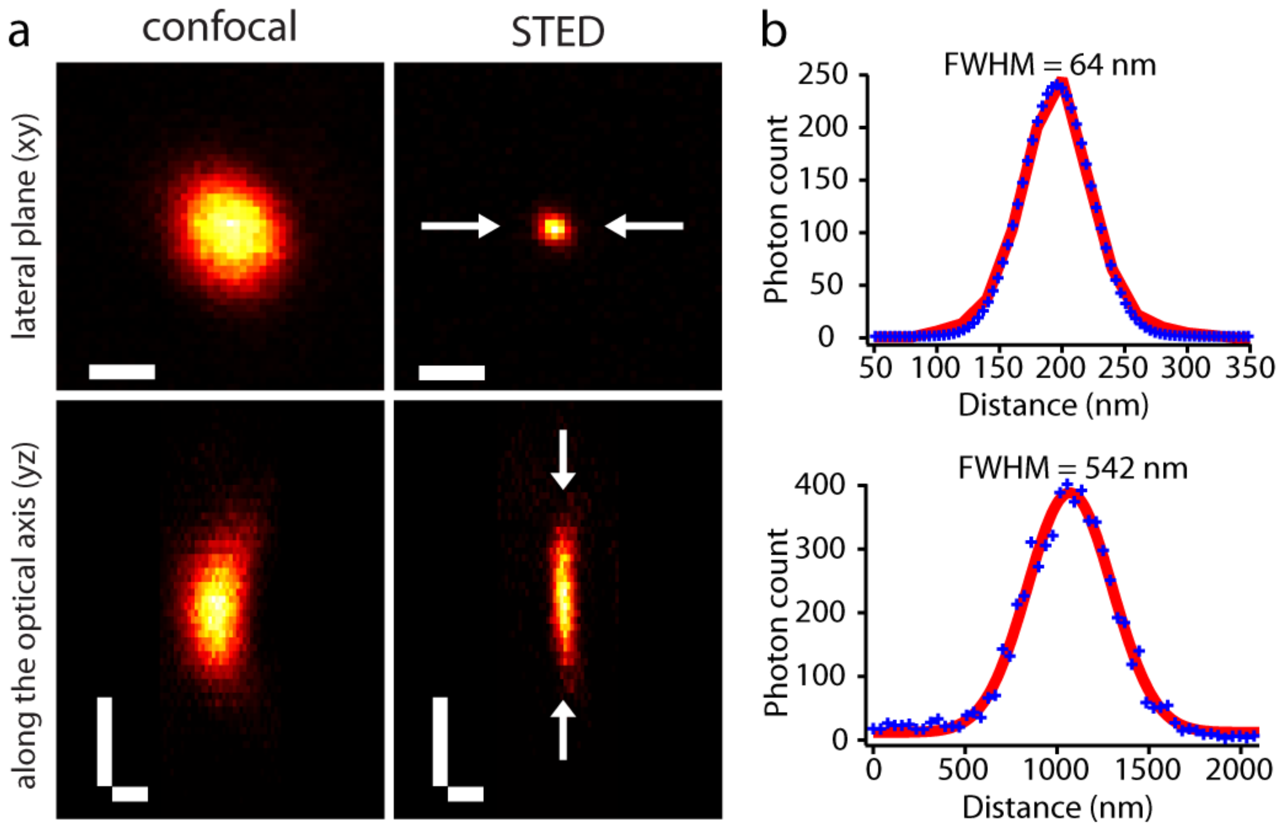

Supplementary Figure 16: Confocal and STED point spread functions

(a) Effective point spread functions (PSFs) of the STED nanoscope used for live *ex vivo* Ca^{2+}-imaging, recorded using 40 nm fluorescent beads (yellow-green Fluorescent Microspheres, Molecular Probes). Confocal (left) and STED (right) PSFs, in the optical (xy) plane (top) and along the optical (yz) axis (bottom). Scale bars: 200 nm (horizontal), 500 nm (vertical).

(b) The STED PSFs here display a FWHM of 64 nm in the optical (xy) plane and of 542 nm along the optical axis (z). Note that these recordings were not taken inside tissue, but mounted on a coverslip.

Supplementary Methods

Potential overestimation of the number of Ca^{2+}-channels measured by application of EGTA

A considerable fraction of Ca^{2+}-channels (estimated here to be up to 50 %) in IHCs is extrasynaptic. Spread of EGTA following iontophoretic application at the AZ might also suppress Ca^{2+}-currents through nearby extrasynaptic channels. If we assume that in IHCs extrasynaptic Ca^{2+}-channels are only found in the ~550 µm² of basolateral membrane (see ref. 5 for details), we arrive at a density of ~1 extrasynaptic channel per µm². Further assuming that EGTA does not spread further than 2 µm from the site of application, we would conclude that no more than ~12 extrasynaptic channels are suppressed by EGTA near the analyzed AZs, resulting in an overestimation of on average ~10%, which would more strongly affect the smaller AZs. However, it appears quite possible that the density of extrasynaptic channels is higher near the AZs, which would result in an even higher overestimation of the number of synaptic Ca^{2+}-channels.

Modeling the contribution of single Ca^{2+}-channels to the optical fluctuation data

The kinetics of the association and dissociation reactions between free Ca^{2+}-ions and the Ca^{2+}-indicator (Fluo-4FF) will affect the properties of the detected fluorescence signal, which is largely dominated by Ca^{2+}-bound Fluo-4FF. In a first step, to assess the degree to which the amplitude of the Ca^{2+}-indicator fluorescence is affected by the duration of the open state of the Ca^{2+}-channel, we used a diffusion–reaction calculator (CalC [http://web.njit.edu/~matveev/calc.html][3]) to simulate synaptic Ca^{2+}-entry into the cytoplasm from a single channel, subsequent diffusion, and reaction with defined buffer species (chosen to match the experimental conditions), as previously described[4]. The simulation volume was cylindrical (3 µm radius and height, with 20 nm edge length of the unitary simulation areas) and we simulated openings of various durations (from 10 µs to 10 ms). The boundary plane harboring the Ca^{2+}-channel was considered to represent the plasma membrane and was therefore set to be reflective for all species. At all other boundaries, the concentration was kept constant at the resting level of the respective species. Because we focused on short durations of Ca^{2+} entry (≤ 10 ms), we disregarded Ca^{2+}-removal mechanisms[5]. The final readout for these simulations was the mean concentration of Ca^{2+}-bound Fluo4-FF in a simulated PSF (centered on the Ca^{2+}-channel and matched to the experimentally determined dimensions of our imaging system), weighted by relative amplitude of the PSF at a given simulated area (Supplementary Fig. 3d). The resulting responses were, in a second step, used to simulate ensembles of Ca^{2+}-bound Fluo4-FF 'transients' from N = 100 individual Ca^{2+}-channels within the PSF of our imaging system, from the onset of the repolarization of the IHC from +53mV to –67mV, i.e. from the onset of the Ca^{2+}-tail current. For these simulations, we assumed a homogenous contribution of all N = 100 Ca^{2+}-channels to the summed signal, and ran 50 repetitions (sweeps), from which we calculated the ensemble mean and variance. For each Ca^{2+}-channel in each sweep, the initial probability to be open (p_o) at the beginning of the sweep (i.e. at the onset of the Ca^{2+}-tail current) was set to 0.9 (slightly higher than the experimentally estimated maximum p_o of 0.82[6]). Then, if a given Ca^{2+}-channel was found to be open at the onset of the simulated sweep (90% probability), its open time was randomly drawn from an exponential probability distribution function with τ = 1.3ms, chosen to be in the range of experimentally measured values for IHC Ca^{2+}-channels[7], and the corresponding Ca^{2+}-bound Fluo4-FF 'transient' chosen. As the steady-state p_o at –67mV is very low, we made the simplifying assumption that a Ca^{2+}-channel will not re-open once it closed. The resulting variance over mean plot was fitted

with a quadratic function to obtain an estimate of the number of Ca^{2+}-channels underlying the ensemble signal[8] (Supplementary Fig. 3c).

Modeling uneven contribution of Ca^{2+}-channels

In addition to potential artifacts arising from bandwidth limitations imposed by the reaction kinetics of the Ca^{2+}-indicator, we expected an effect of an uneven contribution of the individual Ca^{2+}-channels, due to their differential localization within the PSF of our imaging system, which we addressed by modeling (Supplementary Fig. 3d). To this end, we simulated Ca^{2+}-currents for each channel and each sweep in the same way as described above (however, without considering any reaction between free Ca^{2+}-ions and the Ca^{2+}-indicator (Fluo-4FF) as a simplification) and then scaled it by the relative position of the Ca^{2+}-channel with regard to the center of the PSF. Again, we then summed the simulated traces for all Ca^{2+}-channels in each sweep and calculated the ensemble mean and variance for all 50 sweeps (Supplementary Fig. 3e,f).

However, the activity of Ca^{2+}-channels situated completely outside the PSF would still be lost to the imaging process if the Ca^{2+} entering through these channels would not diffuse into the PSF. Considering the larger size of IHC Ca^{2+}-channel clusters (100 – 600 nm, compared to a PSF size of ~280 nm), it appears likely that this method underestimates the number of Ca^{2+}-channels present at the AZ.

Diffusional spread of Ca^{2+}-bound OGB-5N from the AZ

There are two relevant time scales when considering what influence diffusion of the Ca^{2+}-dye or –ions might have on our measurements. First, the diffusion of ions and dye away from the AZ over the course of an image, i.e. over (tens of) milliseconds or until a steady state sets in, and second, the possible movement of the dye during one excitation/detection-cycle (as determined by the temporal separation of the individual laser pulses of ~25ns). The latter effect could conceivably cause a "blurring of the PSF" as excited dye molecules from inside the (effective) focal volume of the STED nanoscope diffuse outside before spontaneously fluorescing.

We performed line-scan recordings of the depolarization-evoked fluorescence increase at IHC AZs in order to measure the speed of the lateral increase of fluorescence away from the AZ. Even though we measured at scan rates of 1.5 kHz, we still could only detect a smaller-sized presynaptic Ca^{2+}-domain during the first line of the scan (< 1ms, see Supplementary Fig. 8a,b). This finding is in line with modeling data indicating that the lateral spread of Ca^{2+}-bound OGB-5N reaches a steady state after as little as 400 µs (Supplementary Fig. 8c). Since this steady state is reached early on during the depolarization, we can assume the Ca^{2+}-domain to be stable during our image acquisition, such that lateral diffusion of the dye does not impair the imaging conditions.

Additionally, diffusional movement of individual excited dye molecules out of the effective focal volume of the STED nanoscope is not expected to have any noticeable impact on the spatial resolution, given the limited time frame. Within the 25 ns elapsing between two consecutive laser pulses, a single dye molecule is expected to move on average as little as 2.3 nm (calculated from $l = \sqrt{t \cdot 2D}$, with l the average diffusion length, t the time available for diffusion [here 25 ns], and D

the diffusion coefficient [here 0.1 μm² ms^{-1}]). Given an effective focal area (during STED measurements) between 50 nm–100 nm, this effect should be entirely negligible.

Estimating [Ca^{2+}]

We performed the calculation of [Ca^{2+}] similar to previously published analysis methods[2,9] which we will explain in detail here. In the case of the dye used in the super-resolution Ca^{2+}-imaging experiments, OGB-5N, we assume that it exists in two distinct states, Ca^{2+}-bound or Ca^{2+}-free (i.e. unbound; Supplementary Fig. 9c,d). We will call these states B (bound) and U (unbound), with the partial fluorescence intensities F_B & F_U, and the partial concentrations $[B]$ & $[U]$. We assume that the fluorescence intensity from each separate state depends linearly on the partial concentration (i.e. there are no nonlinear effects, such as excitation- or detection saturation).

$$F_{\mathrm{B}} = \sigma_{\mathrm{B}}[B],\ F_{\mathrm{U}} = \sigma_{\mathrm{U}}[U] \text{ (Equations 23,24)}$$

Here, σ_{B} and σ_{U} are the coefficients of proportionality for each state. The total fluorescence F will then be the sum of the two individual components $F = F_{\mathrm{B}} + F_{\mathrm{U}}$. For simplicity we further assume the system to be in equilibrium, and the total concentration of the dye $[D]$ = $[B]$ + $[U]$ to be constant.

We furthermore assume that the fluorescence decay of each state can be described by a monoexponential decay with the respective lifetimes $\tau_{\mathrm{B}}, \tau_{\mathrm{U}}$ (Supplementary Fig. 9).

$$F(t) = \alpha_{\mathrm{B}} e^{-\frac{t}{\tau_{\mathrm{B}}}} + \alpha_{\mathrm{U}} e^{-\frac{t}{\tau_{\mathrm{U}}}} \text{ (Equation 25)}$$

Here, α_{B} and α_{U} are the amplitudes of the respective decay curves and t is the time. The fluorescence contribution from each component can be derived simply from the parameters of the exponential decay:

$$F_{\mathrm{B}} = \alpha_{\mathrm{B}} \cdot \tau_{\mathrm{B}},\ F_{\mathrm{U}} = \alpha_{\mathrm{U}} \cdot \tau_{\mathrm{U}} \text{ (Equations 26,27)}$$

In order to calculate the partial concentration of the individual components from the parameters derived from their partial fluorescence, it follows that:

$$\frac{F_{\mathrm{B}}}{F_{\mathrm{U}}} = \frac{\alpha_{\mathrm{B}} \cdot \tau_{\mathrm{B}}}{\alpha_{\mathrm{U}} \cdot \tau_{\mathrm{U}}} = \frac{\sigma_{\mathrm{B}}[B]}{\sigma_{\mathrm{U}}[U]} \text{ (Equation 28)}$$

Or stated differently:

$$\frac{[B]}{[U]} = \frac{\sigma_{\mathrm{U}} \cdot \tau_{\mathrm{B}}}{\sigma_{\mathrm{B}} \cdot \tau_{\mathrm{U}}} \cdot \frac{\alpha_{\mathrm{B}}}{\alpha_{\mathrm{U}}} \text{ (Equation 29)}$$

$$R_{\mathrm{B}} = \frac{[B]}{[B]+[U]} = \frac{\sigma_{\mathrm{U}}[B]}{\sigma_{\mathrm{U}}[B]+\sigma_{\mathrm{U}}[U]} = \frac{\left(\sigma_{\mathrm{U}}/\sigma_{\mathrm{B}}\right) \cdot \sigma_{\mathrm{B}}[B]}{\left(\sigma_{\mathrm{U}}/\sigma_{\mathrm{B}}\right) \cdot \sigma_{\mathrm{B}}[B]+F_{\mathrm{U}}} = \frac{F_{\mathrm{B}}/b}{F_{\mathrm{B}}/b+F_{\mathrm{U}}} \text{ (Equation 30)}$$

With $b := \frac{\sigma_{\mathrm{B}}}{\sigma_{\mathrm{U}}}$ (Equation 15) the brightness correction factor (Supplementary Fig. 10) and R_{B} the fraction of Ca^{2+}-bound dye.

We know that the bound dye is brighter than the unbound dye, both due to longer lifetime as well as due to a higher extinction coefficient. Therefore the fluorescence will be maximal $F_{\max} = \sigma_{\mathrm{B}}[B]_{\max} = \sigma_{\mathrm{B}}[D]$ when the dye is completely in the bound state B and minimal $F_{\min} = \sigma_{\mathrm{U}}[U]_{\max} = \sigma_{\mathrm{U}}[D]$ when completely in the free state U.

As $[D]$ is constant, it follows that:

$$[B] = [D] - [U],\ [U] = [D] - [B] \text{ (Equations 31,32)}$$

Further considering that $F = F_{\mathrm{B}} + F_{\mathrm{U}}$, it follows that:

$$F = F_{\mathrm{B}} + F_{\mathrm{U}} = \sigma_{\mathrm{B}}[B] + \sigma_{\mathrm{U}}([D] - [B]) = (\sigma_{\mathrm{B}} - \sigma_{\mathrm{U}})[B] + F_{\min} \text{ (Equation 33)}$$

$$F = F_{\mathrm{B}} + F_{\mathrm{U}} = \sigma_{\mathrm{B}}([D] - [U]) + \sigma_{\mathrm{U}}[U] = F_{\max} - (\sigma_{\mathrm{B}} - \sigma_{\mathrm{U}})[U] \text{ (Equation 34)}$$

$$=> \frac{F - F_{\min}}{F_{\max} - F} = \frac{(\sigma_{\mathrm{B}} - \sigma_{\mathrm{U}})[B]}{(\sigma_{\mathrm{B}} - \sigma_{\mathrm{U}})[U]} = \frac{[B]}{[U]} \text{ (Equation 35)}$$

$$=> \frac{F - F_{\min}}{F_{\max} - F_{\min}} = \frac{(\sigma_{\mathrm{B}} - \sigma_{\mathrm{U}})[B]}{(\sigma_{\mathrm{B}} - \sigma_{\mathrm{U}})[D]} = \frac{[B]}{[D]} = R_{\mathrm{B}} \text{ (Equation 36)}$$

In a state of equilibrium the law of mass action holds:

$$K_{\mathrm{D}} = \frac{[U][Ca^{2+}]}{[B]} \text{ (Equation 37)}$$

$$=> \frac{[Ca^{2+}]}{K_{\mathrm{D}}} = \frac{[B]}{[U]} = \frac{F - F_{\min}}{F_{\max} - F} \text{ (Equation 38)}$$

$$=> [Ca^{2+}] = K_{\mathrm{D}} \frac{F - F_{\min}}{F_{\max} - F} \text{ (Equation 39)}$$

Furthermore it follows that:

$$1 + \frac{[U]}{[B]} = 1 + \frac{K_{\mathrm{D}}}{[Ca^{2+}]} <=> \frac{[B] + [U]}{[B]} = \frac{[Ca^{2+}] + K_{\mathrm{D}}}{[Ca^{2+}]} \text{ (Equation 40)}$$

From this follows:

$$\frac{[Ca^{2+}]}{[Ca^{2+}] + K_{\mathrm{D}}} = \frac{[B]}{[B] + [U]} = \frac{[B]}{[D]} = R_{\mathrm{B}} \text{ (Equation 41)}$$

And also:

$$\frac{[Ca^{2+}]}{[Ca^{2+}] + K_{\mathrm{D}}} = \frac{[B]}{[D]} = \frac{\sigma_{\mathrm{B}}[B]}{\sigma_{\mathrm{B}}[D]} = \frac{F_{\mathrm{B}}}{F_{\max}} \text{ (Equation 42)}$$

$$=> F_{\mathrm{B}} = F_{\max} \cdot \frac{[Ca^{2+}]}{[Ca^{2+}] + K_{\mathrm{D}}} \text{ (Equation 43)}$$

Considering a Ca^{2+}-independent background signal

Although we never observed any noticeable Ca^{2+}-independent background originating from the OGB-5N *in vitro*, a Ca^{2+}-independent background was present in the environment of the IHCs. Here, we observed fluorescence signals both in the fast and slow channel that persisted even in Ca^{2+}-saturated or -free environments, limiting the observable dynamic range of the dye to ~2-4% < R_B < 60%, i.e. showing several percent of bound dye where no Ca^{2+} should be present, and over 40% of free dye in an environment which should saturate the dye. In order to correctly interpret the data from the experiments in IHCs, we adapted our equations to incorporate an additional (background) signal from a Ca^{2+}-independent portion of the dye, which can be separated into a slow and a fast component.

$$\begin{aligned} F' &:= \underbrace{(F_\mathrm{B} + F_\mathrm{U})}_{Ca^{2+}-sensitive} + \underbrace{(X_\mathrm{B} + X_\mathrm{U})}_{Ca^{2+}-independent} \\ &= \underbrace{(F_\mathrm{B} + X_\mathrm{B})}_{slow} + \underbrace{(F_\mathrm{U} + X_\mathrm{U})}_{fast} \\ &=: F'_\mathrm{B} + F'_\mathrm{U} \end{aligned} \qquad \text{(Equation 44)}$$

With F' the measured fluorescence (including Ca^{2+}-insensitive signal), F'_B and F'_U the total fluorescence signal (as provided by the fitting algorithm) in the bound (slow) and unbound (fast) channel, respectively, X_B, X_U the Ca^{2+}-independent signal and F_B, F_U the (desired) Ca^{2+}-sensitive signal.

Assuming there was no Ca^{2+}-insensitive background signal, we could calculate $[Ca^{2+}]$ as derived above:

$$\frac{F_\mathrm{B}}{F_\mathrm{max} - F_\mathrm{B}} = \frac{\sigma_\mathrm{B} B}{\sigma_\mathrm{B} D - \sigma_\mathrm{B} B} = \frac{B}{D-B} = \frac{B}{U} = \frac{[Ca^{2+}]}{K_\mathrm{eff}} \qquad \text{(Equation 45)}$$

If we now assume that at resting potential inside the IHC, the free $[Ca^{2+}]$ is in the range of 50 nM[4,10], then the Ca^{2+}-sensitive dye should be (nearly) completely in the free state, with only marginal signal from the bound state. If we now observe the difference between the resting and the stimulated state, we obtain:

$$\Delta F'_\mathrm{B} = F'_\mathrm{B,stim} - F'_\mathrm{B,rest} = (F_\mathrm{B,stim} - X_\mathrm{B}) - (\underbrace{F_\mathrm{B,rest}}_{=0} - X_\mathrm{B}) = F_\mathrm{B} = \Delta F_\mathrm{B} \qquad \text{(Equation 46)}$$

So by observing the difference between the stimulated and resting states, we can eliminate the slow (Ca^{2+}-independent) background signal.

Furthermore, as in the resting state almost all of the Ca^{2+}-sensitive dye is unbound, we can take the fluorescence from the fast channel to estimate F_{min}, and, by extension, F_{max}. From our *ex vivo* calibration measurements in IHCs we observed fluorescence in the fast channel corresponding to 40% of the total dye, even at saturating Ca^{2+}-values. We can therefore assume that in the resting state Ca^{2+}-sensitive dye corresponds to 60% of the total signal:

$$F'_\mathrm{U,rest} = \underbrace{F_\mathrm{U,rest}}_{=F_\mathrm{min}} + \underbrace{X_\mathrm{U}}_{=0.4 \cdot F'_\mathrm{U,rest}} \qquad \text{(Equation 47)}$$

$$F_\mathrm{min} = \underbrace{0.6}_{:=r_\mathrm{max,bound}} \cdot F'_\mathrm{U,rest} \qquad \text{(Equation 48)}$$

With these two equations, we can eliminate the Ca^{2+}-insensitive background and calculate the $[Ca^{2+}]$ according to the earlier derived equations:

$$[Ca^{2+}] = K_{\text{eff}} \cdot \frac{F_{\text{B}}}{F_{\text{max}} - F_{\text{B}}} = K_{\text{eff}} \cdot \frac{\Delta F'_{\text{B}}}{F_{\text{min}} \cdot b - \Delta F'_{\text{B}}} = K_{\text{eff}} \cdot \frac{\Delta F'_{\text{B}}}{F'_{\text{U,rest}} \cdot b \cdot r_{\text{max,bound}} - \Delta F'_{\text{B}}} \text{ (Equation 49)}$$

In the case of STED measurements, the recorded signal in the fast channel needs to be adjusted by the STED efficiency factor s to correct for the different STED efficiencies of the fast and slow dye component:

$$[Ca^{2+}] = K_{\text{eff}} \cdot \frac{\Delta F'_{B}}{F'_{\text{U,rest}} \cdot b \cdot r_{\text{max,bound}} \cdot s - \Delta F'_{\text{B}}} \text{ (Equation 50)}$$

For the *ex vivo* measurements in IHCs, F_{min} was calculated not pixel by pixel, but by averaging over each pixel in the raster scan, thus minimizing the noise.

Comparison of lifetime-based calculation of $[Ca^{2+}]$ with a fluorescence-increase based method

To discern whether our lifetime method produced comparable results to the established method of measuring the fluorescence increase upon calcium binding, we calculated the ratio of bound and unbound dye both using our lifetime method (including the fitting routine) and using only fluorescence increase for confocal measurements. According to Equation 36,

$$\frac{F - F_{\text{min}}}{F_{\text{max}} - F_{\text{min}}} = \frac{[B]}{[D]} = R_{\text{B}} = \frac{F_{\text{B}}/b}{F_{\text{B}}/b + F_{\text{U}}} \text{ (Equation 51)}$$

we plotted the percentage of bound dye calculated via lifetime method against the ratio $\frac{F - F_{\text{min}}}{F_{\text{max}} - F_{\text{min}}}$ (Supplementary Fig. 15a). As can be seen, the data forms a near perfect diagonal, with a linear fit revealing a slope of 1.1, showing that both methods estimate comparable amounts of bound dye and, by extension, amounts of free $[Ca^{2+}]$.

The percentage of Ca^{2+}-bound dye as estimated from the lifetime method is directly dependent on the brightness correction factor, the fluorescence increase ratio, however, is not. Therefore, we can use this identity to verify and, if necessary, readjust the brightness correction factor. As the data shows, though, the brightness value of 28.02 matched the data quite well (Supplementary Fig. 15a).

Now that we have verified the lifetime method, we can use the confocal measurements as a scale by which to judge the data recorded using STED, for which the calculations are more difficult and which require an additional correction factor. In Supplementary Fig. 15b we plotted the percentage of slow dye obtained from the STED measurements against the data from the confocal experiments. It can be seen, that up until an amount of about 30%—40% of bound dye, both the STED and confocal experiments reveal nearly identical data points *in vitro*. As the data recorded in the measurements in IHCs was always in the range of 1—20% of bound dye, this is precisely inside of this range. The $[Ca^{2+}]$ determined by STED measurements should therefore match the results from confocal recordings

For values higher than 40% of bound dye, the curve deviates from the diagonal and instead the STED experiments overestimate the amount of free dye (Supplementary Fig. 15b). This is probably due to the fact that the fit ignores the initial region immediately following the excitation pulse (“blanking”,

Supplementary Fig. 12) in order to omit any fluorescence decrease caused by the STED deactivation, which could erroneously be interpreted as a contribution by the short lifetime of the unbound dye. With increasing [Ca^{2+}], the diminishing fluorescence from the free dye is being increasingly overwhelmed by photons from the very bright, bound dye. As the contribution to the fast decay becomes smaller and smaller, the reliability of fitting the remnant decreases, leading to a persistent overestimation of the free dye. Therefore, this method is not very accurate in these ranges. The STED data reaches maximal values for a percentage of bound dye of around 75%. In order to improve the estimate for values of Ca^{2+}-bound dye higher than 40%, an alternative method of fitting and correction needs to be applied.

Supplementary References

1. Wong, A. B. *et al.* Developmental refinement of hair cell synapses tightens the coupling of Ca2+ influx to exocytosis. *EMBO J* **33,** 247–264 (2014).
2. Wilms, C. D. & Eilers, J. Photo-physical properties of Ca2+-indicator dyes suitable for two-photon fluorescence-lifetime recordings. *Journal of microscopy* **225,** 209–213 (2007).
3. Matveev, V., Sherman, A. & Zucker, R. S. New and Corrected Simulations of Synaptic Facilitation. *Biophysical Journal* **83,** 1368–1373 (2002).
4. Frank, T., Khimich, D., Neef, A. & Moser, T. Mechanisms contributing to synaptic Ca2+ signals and their heterogeneity in hair cells. *Proc Natl Acad Sci USA* **106,** 4483–4488 (2009).
5. Kennedy, H. J. Intracellular calcium regulation in inner hair cells from neonatal mice. *Cell Calcium* **31,** 127–136 (2002).
6. Brandt, A., Khimich, D. & Moser, T. Few CaV1.3 Channels Regulate the Exocytosis of a Synaptic Vesicle at the Hair Cell Ribbon Synapse. *J. Neurosci.* **25,** 11577–11585 (2005).
7. Zampini, V. *et al.* Elementary properties of CaV1.3 Ca2+ channels expressed in mouse cochlear inner hair cells. *The Journal of Physiology* **588,** 187–199 (2010).
8. Steffan, R. & Heinemann, S. H. Error estimates for results of nonstationary noise analysis derived with linear least squares methods. *Journal of Neuroscience Methods* **78,** 51–63 (1997).
9. Hirshfield, K. M., Toptygin, D., Packard, B. S. & Brand, L. Dynamic Fluorescence Measurements of Two-State Systems: Applications to Calcium-Chelating Probes. *Analytical Biochemistry* **209,** 209–218 (1993).
10. Beutner, D. & Moser, T. The presynaptic function of mouse cochlear inner hair cells during development of hearing. *J Neurosci* **21,** 4593 (2001).
11. Faas, G. C., Karacs, K., Vergara, J. L. & Mody, I. Kinetic properties of DM-nitrophen binding to calcium and magnesium. *Biophys. J* **88,** 4421–4433 (2005).
12. Morgado-Valle, C., Beltran-Parrazal, L., DiFranco, M., Vergara, J. L. & Feldman, J. L. Somatic Ca2+ transients do not contribute to inspiratory drive in preBötzinger Complex neurons. *J Physiol* **586,** 4531–4540 (2008).
